# Trends in otolaryngology publications: a 9-year bibliometric analysis of articles published in Journal of Otolaryngology—Head and Neck Surgery

**DOI:** 10.1186/s40463-022-00619-0

**Published:** 2023-02-16

**Authors:** Keshinisuthan Kirubalingam, Agnieszka Dzioba, Yvonne Chan, M. Elise Graham

**Affiliations:** 1 Queen’s University School of Medicine, Kingston, Canada; 2 Department of Otolaryngology–Head and Neck Surgery, Children’s Hospital at London Health Sciences Centre, and Schulich School of Medicine and Dentistry, University of Western Ontario, 800 Commissioners Road E, Box 5010, London, ON N6A 5W9 Canada; 3 Department of Otolaryngology–Head and Neck Surgery, St Michael’s Hospital, University of Toronto, Toronto, ON Canada

**Keywords:** Otolaryngology, Bibliometrics, Evidence-based medicine, International collaborations

## Abstract

**Background:**

The advancement of Otolaryngology—Head and Neck Surgery (OHNS) as a specialty relies on excellence in research. The *Journal of Otolaryngology-Head and Neck Surgery* is an open access, peer-reviewed journal publishing on all aspects and subspecialities of OHNS. It is the official journal of the Canadian Society of Otolaryngology—Head and Neck Surgery. This study aims to analyze bibliometric trends in authorships and institutional contributions within the *Journal of Otolaryngology-Head and Neck Surgery* over a 9-year period.

**Methods:**

All research articles published online in the journal were analyzed from 2013 to the end of 2021. The professional designation of all authors was recorded along with the article type, article category, institutional affiliations and international collaborations. Cochran–Armitage trend tests were used to assess the change in proportion over time between years and groups.

**Results:**

Of the 603 articles, 20 were excluded as they represented correspondence or corrections, or author identity could not be determined. 583 articles with 3409 total authors were included. Number of first authors with a Doctor of Medicine (MD) degree decreased from 90.2 to 85.3% (*P* = 0.165). Sub-group analysis of non-MD first authors demonstrated a significant increase in medical students as first authors from 1.6 to 11.8% (*P* = 0.008). Senior author degree demonstrated a significant increase in MD degree from 96.7 to 98.5% (*P* = 0.002). Analysis of article categories demonstrated a significant decrease in education and head and neck surgery related articles from 8.2 to 2.9% (*P* = 0.032) and 44.3 to 29.4% (*P* = 0.028) respectively. Pediatric otolaryngology articles increased significantly from 0 to 5.9% (*P* < 0.0001). Systematic and scoping reviews significantly increased, from 3.3 to 10.3% (*P* = 0.015) and original research significantly decreased from 83.6 to 82.4% (*P* < 0.0001). There was a significant decrease in Canadian/international collaborations from 14.3 to 4.7% (*P* = 0.037). There was a significant increase in international first and senior authors, from 23.0 to 36.8% (*P* = 0.008) and 19.7 to 38.2% (*P* = 0.002) respectively.

**Conclusion:**

The landscape of the journal is evolving with increased representation of non-MDs and international authors along with content that reflects higher level of scientific evidence. Future studies should characterize trends in other Otolaryngology journals to understand the research trajectory within the field.

**Graphical abstract:**

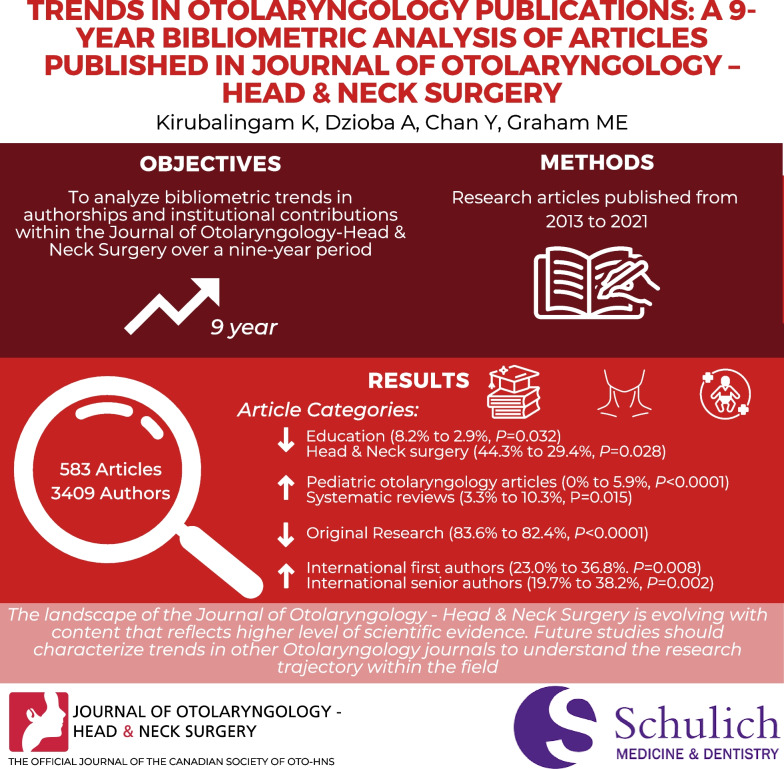

## Background

The practice of evidence-based medicine (EBM) is critical to optimize patient care [1]. It relies on the best available research to inform clinical and surgical decision-making. Otolaryngology—Head and Neck Surgery (OHNS) is a rapidly-changing and highly innovative field that is fueled by ongoing research [2]. With incorporation of EBM, OHNS has transitioned from a purely apprenticeship model, guided by anecdotal evidence and expert opinion, to one where an evidence-based approach represents the expected standard of care [2].

The quality of research articles published by a prominent specialty-specific journal is a key metric of the growing contributions to knowledge creation [3]. Bibliometrics is the process of analyzing the content and citations of journal articles to quantify trends in publication type, topic area, institutions of origin and dissemination of published data [4]. In OHNS, previous bibliometric studies indicate a considerable increase in the quantity and quality of research in the international arena [5, 6]. However, within the Canadian landscape, Gurberg et al., demonstrated a decrease in Canadian authored papers in Otolaryngology journals over a 4-year period from 2008 to 2012 [7].

The *Journal of Otolaryngology-Head and Neck Surgery* is an open access, peer-reviewed journal publishing on all aspects and subspecialities of OHNS, including pediatric and geriatric otolaryngology, rhinology and anterior skull base surgery, otology/neurotology, facial plastic and reconstructive surgery, head and neck oncology, maxillofacial rehabilitation, as well as a broad range of related topics [8]. It is the official journal of the Canadian Society of Otolaryngology—Head and Neck Surgery. There is currently no bibliometric Canadian OHNS study exploring the trends of publications in this national journal. A detailed analysis of authorship contributions, literature appraisal and article classifications can help identify major stakeholders in the Canadian academic OHNS landscape. Such information can guide aspiring OHNS scientists in future research and training endeavors. Most importantly, this knowledge can help foster local and international collaborations by showcasing existing work. Furthermore, data on thematic contributions can highlight contemporary topics and subspecialities with increased research productivity. Finally, data collected to highlight these factors since the online inception of the journal can be used as a starting point to track evolving trends in the future.

Therefore, the objective of the current study is to examine trends of OHNS publications in the Canadian-based *Journal of Otolaryngology-Head and Neck Surgery* over a 9-year period. We analyzed Canadian and international collaborations along with authorship and article details. This study is the first to use a comprehensive dataset to elucidate publication trends in the *Journal of Otolaryngology-Head and Neck Surgery*.

## Methods

### Data source/author identification

The online archive of the *Journal of Otolaryngology-Head and Neck Surgery* was utilized to identify articles published from January 2013 through December 2021. January 2013 was chosen as this was the first year the Journal established online open access articles. Data extraction was performed by a single author (K.K.) independently. Article title, category, type, publication date, first and senior author name, degree, country of origin and international collaborations were identified. Articles were categorized into Education, Facial Plastics, General OHNS, Head and Neck, Laryngology, Otology, Pediatric OHNS, Rhinology and “other” (did not fit in any previously listed category). Article types identified include original research, systematic/scoping review, narrative review, case report and case series. First author degrees were categorized into Doctor of Medicine (MD) and non-MD, with non-MD criteria including Bachelor’s degree (BSc), Master’s degree (MSc), current medical student, Doctor of Dental Surgery (DDS), Doctor of Philosophy (Ph.D.) and combined DDS/Ph.D. Authors with multiple degrees in addition to MD such as MD/Ph.D., MD/MSc were included in the MD category. For articles that only listed the authors’ first initials, attempts were made to find the full first name through additional publication databases (e.g., PubMed, MEDLINE) and Internet search engines (e.g., Google). An Internet search was conducted to obtain all authors’ details using institutional website information and publicly available social media information (i.e., LinkedIn). Current institution or practice affiliation was also collected for all authors along with country of origin. Articles with only a single author were assigned to the first author cohort and excluded from the senior author cohort. Any author whose identity could not be determined, i.e., name or affiliation, was considered “unknown,” and the article was omitted from analysis.

### Statistical analyses

A Microsoft Excel database (Microsoft Corp., Redmond, WA) was utilized for data collection and storage. Data was analyzed using SPSS, version 27 and Excel. Cochran-Armitage trend test was used to assess the change in proportions of article type/category, author degrees and degree of international collaboration over time. A *P* value of < 0.05 was considered significant.

All data used in this study were publicly available and de-identified, therefore the study was exempt from institutional ethics board review, as per the Tri-Council Policy Statement Article 2.2 [9].

## Results

**Fig. 1:**
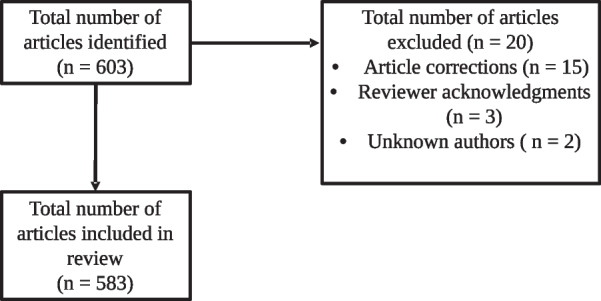
Flow chart of study selection

A total of 603 articles were identified, of which 20 were excluded because they were corrections of previous articles, the authors were unknown, or were not scientific articles (i.e., reviewer acknowledgements written from the editorial board). Of the 583 articles analyzed, 3409 total authors were identified (Fig. 1).

**Fig. 2:**
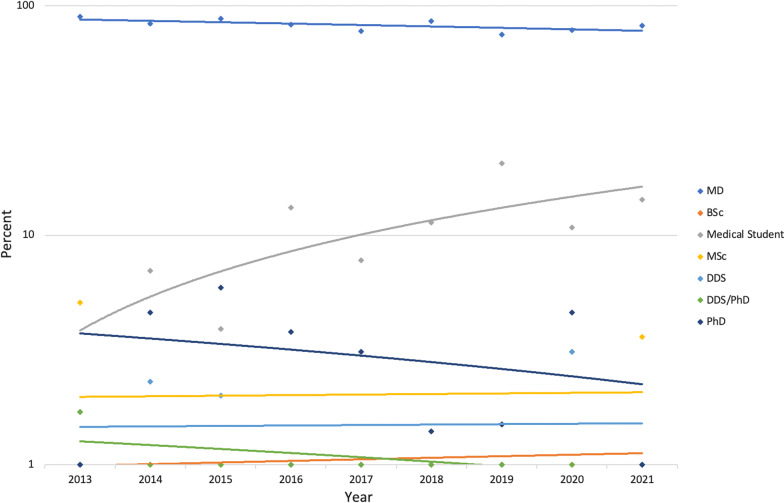
Proportion of first author degree by year. The proportion of first authors with MD degrees decreased over the study period but the change was not significant (*P* = 0.165). The increase noted in medical student first authors was statistically significant (*P* = 0.008)

Analysis of first author degree demonstrated a non-significant decrease in MDs from 90.2% in 2013 to 85.3% in 2021 (*P* = 0.165). Non-MD first author designation increased non-significantly from 9.8% in 2013 to 14.7% in 2021 (*P* = 0.165). Sub-group analysis of non-MD first authors demonstrated a significant increase in first authors who are medical students from 1.6% in 2013 to 11.8% in 2021 (*P* = 0.008). There was no change noted in those with BSc (*P* = 0.504), DDS (*P* = 0.826) and Ph.D. (*P* = 0.687). First author MSc designation decreased non-significantly from 4.9% in 2013 to 2.9% in 2021 (*P* = 0.991) along with combined DDS/Ph.D. from 1.6% in 2013 to 0% in 2021 (*P* = 0.096) (Fig. 2).

**Fig. 3:**
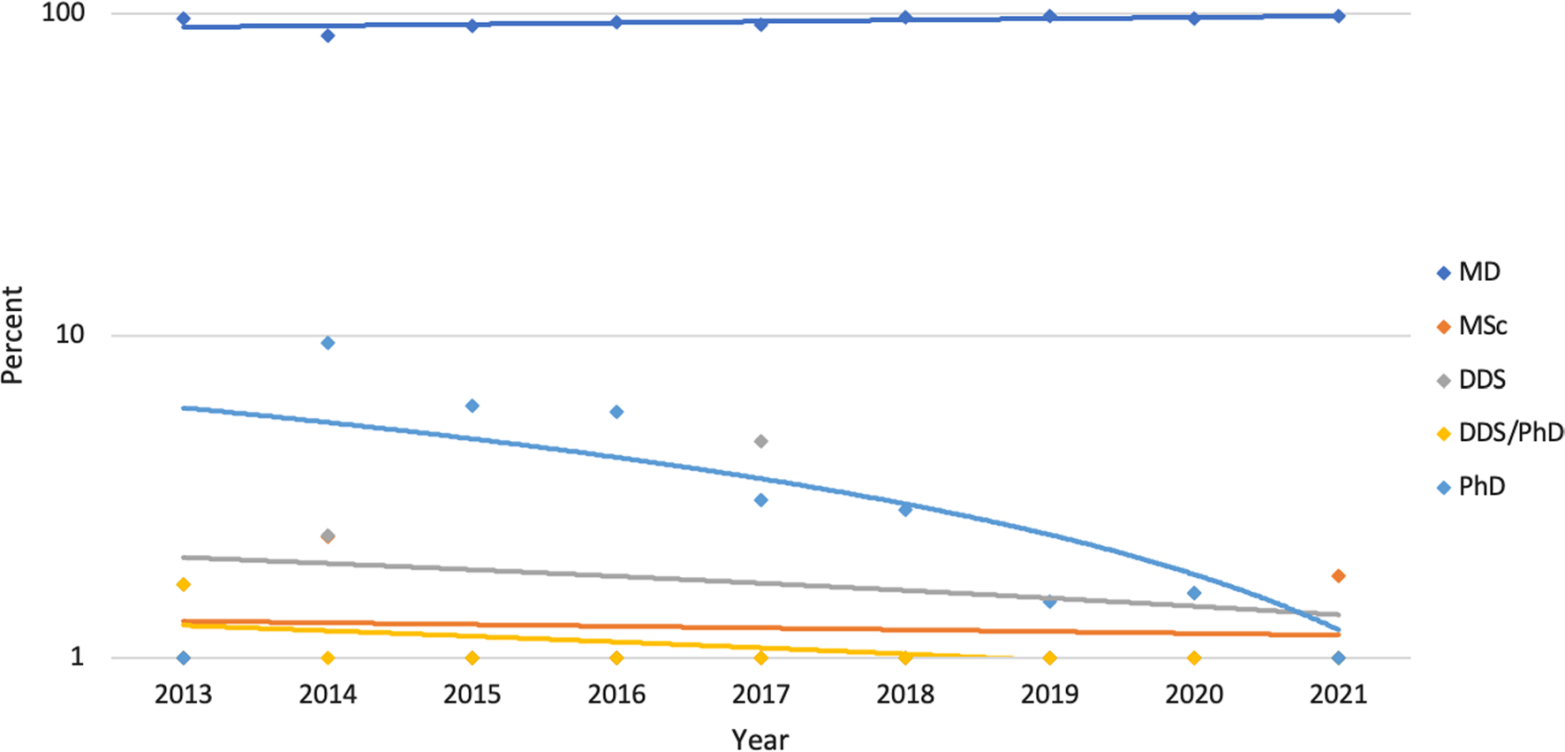
Proportion of senior author degree by year. Statistically significant changes in senior authors over time include an increase in MDs (*P* = 0.002) and decrease in Ph.D. senior authors (*P* = 0.0012)

Analysis of senior author degree demonstrated a statistically significant increase in MDs from 96.7% in 2013 to 98.5% in 2021 (*P* = 0.002). Non-MD senior author designation significantly decreased from 3.3% in 2013 to 1.5% in 2021 (*P* = 0.002). Sub-group analysis of non-MD senior authors demonstrates no change in those with a Ph.D. (*P* = 0.012). Senior authors with MSc designation increased non-significantly from 0% in 2013 to 1.50% in 2021 (*P* = 0.901). Senior authors with DDS and combined DDS/Ph.D. decreased non-significantly from 1.6% in 2013 to 0% in 2021 (*P* = 0.181, *P* = 0.096 respectively) (Fig. 3).

**Fig. 4:**
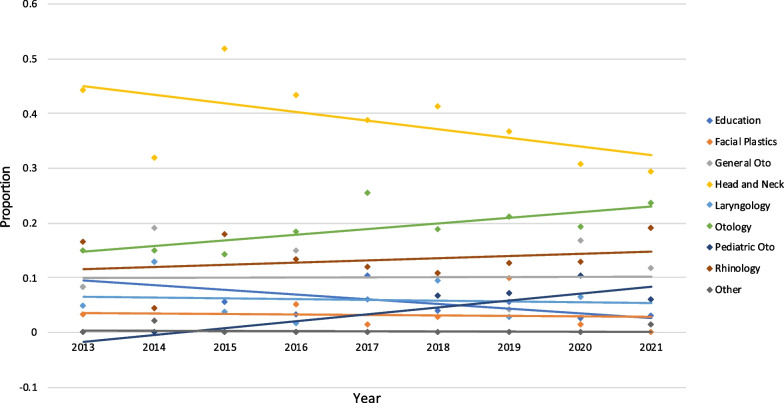
Proportion of article categories by year. Statistically significant decreases over time were seen in articles in education (*P* = 0.032) and head and neck surgery (*P* = 0.028), while a statistically significant increase in pediatric OHNS articles was seen (*P* < 0.0001)

Analysis of article categories demonstrated a statistically significant decrease in articles focused on education from 8.2% in 2013 to 2.9% in 2021 (*P* = 0.032), and those focused on head and neck surgery from 44.3% in 2013 to 29.4% in 2021 (*P* = 0.028). Articles in Pediatric OHNS increased significantly, from 0% in 2013 to 5.9% in 2021 (*P* < 0.0001). Facial plastic surgery articles decreased non-significantly from 3.3% in 2013 to 0% in 2021 (*P* = 0.691). Articles in the following categories increased in proportion between 2013 and 2021: general otolaryngology, from 8.2 to 11.8% (*P* = 0.759); laryngology, from 4.9 to 5.9% (*P* = 0.848); otology, from 14.8 to 23.5% (*P* = 0.129); rhinology, from 16.4 to 19.1% (*P* = 0.578); and “other” from 0 to 1.5% (*P* = 0.879), however none of these changes were statistically significant (Fig. 4).

Analysis of article type demonstrated a significant increase in systematic and scoping review articles from 3.3% in 2013 to 10.3% in 2021 (*P* = 0.015). There was a statistically significant decrease in original research from 83.6% in 2013 to 82.4% in 2021 (*P* < 0.0001). Article types that decreased non-significantly from 2013 to 2021 include case reports from 4.9 to 2.9% (*P* = 0.162), case series from 3.3 to 1.5% (*P* = 0.244), and narrative reviews from 4.9 to 2.9% (*P* = 0.182) (Table 1).

**Table 1:** Proportion of article types from 2013 to 2021

Article type	2013	2014	2015	2016	2017	2018	2019	2020	2021	*P*
Case report	3/61 (4.9%)	1/47 (2.1%)	3/56 (5.4%)	5/60 (8.3%)	1/67 (1.5%)	3/75 (4.0%)	8/71 (11.3%)	10/78 (12.8%)	2/68 (2.9%)	0.162
Case series	2/61 (3.3%)	0/47 (0%)	1/56 (1.8%)	1/60 (1.7%)	1/67 (1.5%)	3/75 (4.0%)	3/71 (4.2%)	5/78 (6.4%)	1/68 (1.5%)	0.244
Narrative review	3/61 (4.9%)	2/47 (4.3%)	0/56 (0%)	1/60 (1.7%)	2/67 (3.0%)	4/75 (5.3%)	2/71 (2.8%)	9/78 (11.5%)	2/68 (2.9%)	0.182
Original research	51/61 (83.6%)	43/47 (91.5%)	49/56 (87.5%)	50/60 (83.3%)	61/67 (91.0%)	59/75 (78.7%)	52/71 (73.2%)	47/78 (60.3%)	56/68 (82.4%)	**< 0.0001**
Systematic/scoping review	2/61 (3.3%)	1/47 (2.1%)	3/56 (5.4%)	3/60 (5.0%)	2/67 (3.0%)	6/75 (8.0%)	6/71 (8.7%)	7/78 (9.0%)	7/68 (10.3%)	**0.015**

Bolded values are statistically significant

Of the 435 articles with Canadian first or senior authors, 60 (13.8%) had collaborators from another country. There was a significant decrease in Canadian-International collaboration from 14.3% in 2013 to 4.7% in 2021 (*P* = 0.037). Countries with which Canadian authors collaborated included: United States (n = 24), Saudi Arabia (n = 10), United Kingdom (n = 7), Australia (n = 3), Japan (n = 2), Korea (n = 2), Singapore (n = 2), Egypt (n = 1), Finland (n = 1), France (n = 1), Germany (n = 1), Guyana (n = 1), New Zealand (n = 1), Switzerland (n = 1), Thailand (n = 1), Uganda (n = 1).

**Fig. 5:**
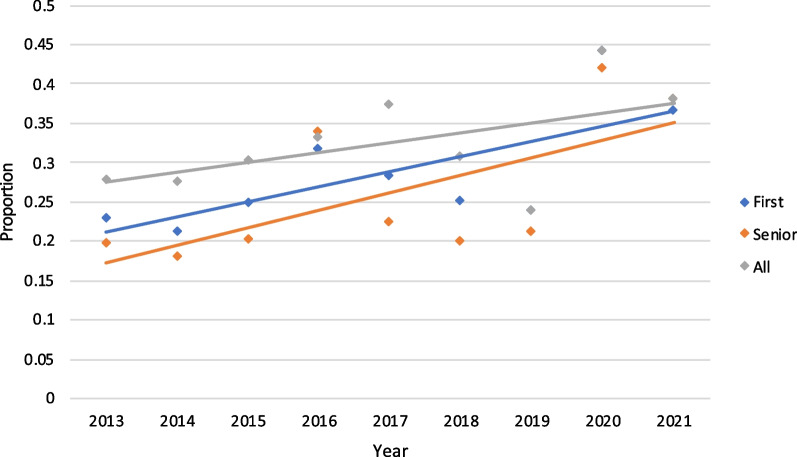
Proportion of international first, senior and combined authors by year. The change in overall international authorship did not change significantly over time, though there was a statistically significant increase in first and senior authors arising from locations outside Canada (*P* = 0.008 and *P* = 0.002, respectively)

**Fig. 6:**
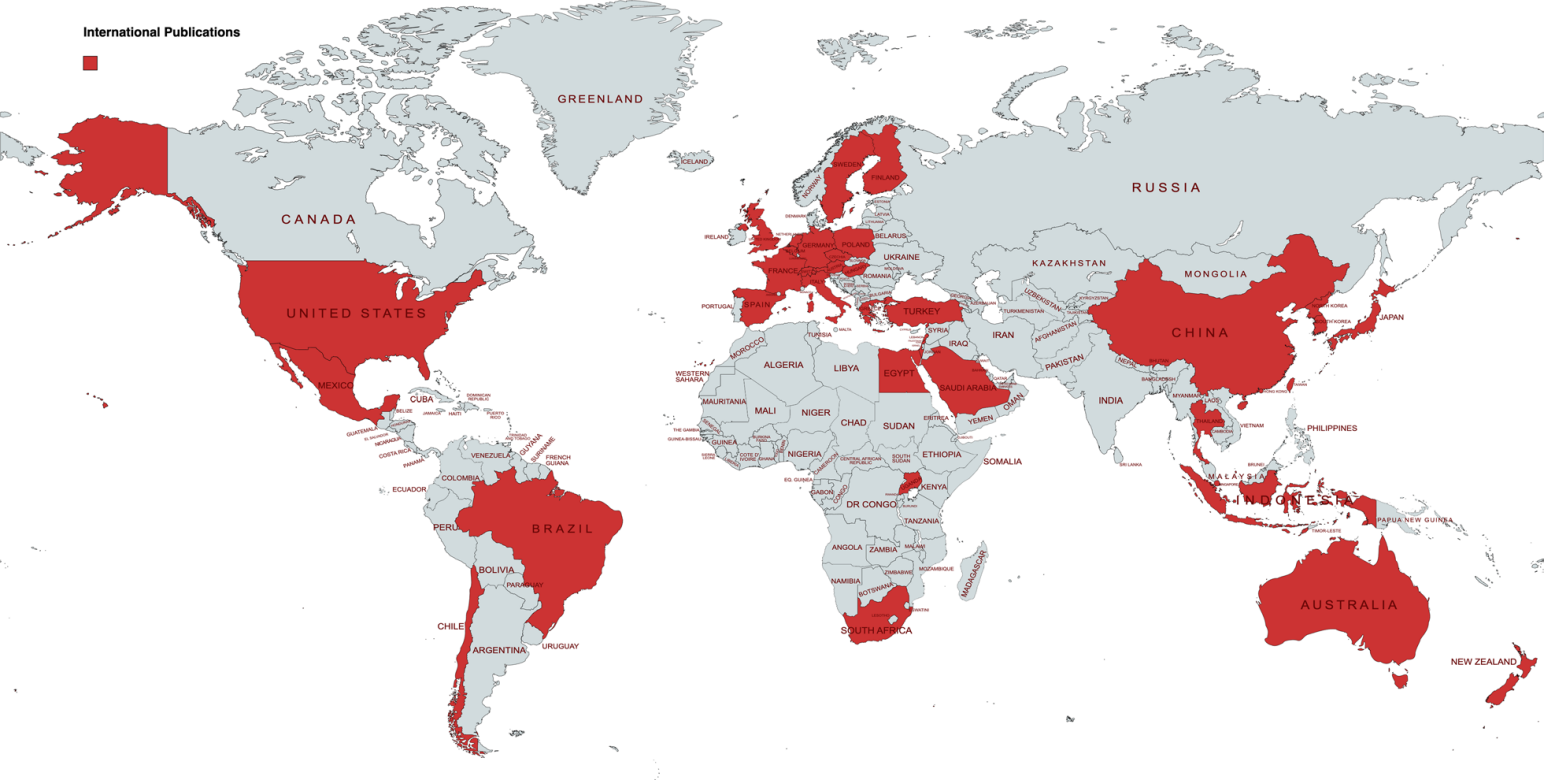
Countries with international publications to the journal over the 9-year period. International authors arose in the highest proportions from China (10.98%) and the United States (8.40%)

When examining articles with first author originating outside Canada, there was a statistically significant increase in international representation, from 23.0% in 2013 to 36.8% in 2021 (*P* = 0.008). Similarly, there was also a statistically significant increase in international senior author representation from 19.7% in 2013 to 38.2% in 2021 (*P* = 0.002). Combined international author representation (including first, middle and senior authors) revealed a non-significant increase from 27.9% in 2013 to 38.2% in 2021 (*P* = 0.097) (Fig. 5). Articles published by authors from non-Canadian countries had highest representation from China (10.98%) and United States (8.40%) between 2013 and 2021. (Fig. 6).

## Discussion

This bibliometric study aims to characterize the trends of authorship and institutional contributions within the Canadian *Journal of Otolaryngology-Head and Neck Surgery.* Our findings indicate changes in authorship contributions with increased MD senior author representation. Thematic content analysis revealed increase in review literature in comparison to original research articles. Lastly, there is a significant increase in international participation highlighting the expansion of the journal within the global research landscape.

### Trends in first and senior author designations

Our findings demonstrate a significant decrease in MD first authors over the 9-year period with an increase in current medical students as first authors. OHNS residency has historically been competitive, with the 2021 Canadian Resident Matching Service data placing it as the top competitive specialty with a supply demand ratio of 0.49 in the first match iteration [10]. Furthermore, a 2020 study analyzing factors affecting Canadian medical students’ success in the residency match identified that there is a prevailing belief among students that enhancing one’s curriculum vitae with research productivity will increase the likelihood of a successful match [11]. It also demonstrated that the number of research activities for those pursuing a competitive discipline was higher than those for less competitive disciplines [11]. Additionally, US studies indicate that productive research experience appear to be a strong predictor of a successful match in OHNS [12].

In terms of senior authorship, there was a significant increase in authors with MD over the 9-year period with subsequent decrease in non-MD author representation. This may suggest that clinical researchers are increasingly generating high quality work for publication. Furthermore, as the number of publications held is often a significant factor for securing research funding and career advancement, medical professionals may be increasingly motivated to attain senior authorship in scientific journals to enhance professional growth. Non-MD authors may be selecting different journals that align more specifically with their research focus, or may face barriers when submitting to this journal. Previous studies have shown that basic science presentations at annual meetings did not necessarily have a greater publication rate compared to clinical work [13].

### Trends by article category

With respect to article categories, we note a significant decrease in education articles published in the journal over the 9 years examined. High quality education research serves to enhance clinical practices and careers of current trainees and surgical educators. A 2016 bibliometric analysis identified that publications in OHNS education increased rapidly from 2000 to 2015 with education articles being published more frequently in higher-impact OHNS journals [14]. Furthermore, OHNS education research can also be published in education-focused (non-OHNS) journals with high impact factors. It may be that these education articles are being increasingly published in different or dedicated journals. Funding allocations for medical education research may also play a role in impacting research quantity and quality. Future work should emphasize the importance of quality education research with regard to training the next generation of OHNS surgeons in a time of increasing complexity of surgical techniques and technologies.

Head and Neck Surgery was another area in which publications significantly decreased over time. The approach to head and neck oncology is complex with multidisciplinary experts involved in the care of patients [15]. Despite the rapid advancements in the field, there is a pressing need for clinical and translational research to advance treatment outcomes [16]. Currently, there is a shift towards understanding the cancer biology at a molecular level to identify biomarkers and individualize therapy [17]. However, some of the caveats of translational research include the resource and time required for basic science research and the associated challenges of transforming the findings into clinical trials [18]. Currently, a fifth of surgical trials in general are abandoned and less than half published [19]. The length of time along with the high attrition rate could potentially reflect the decrease in head and neck research within the journal. However, there also might be a shift in preference for other journals with higher impact factors, or the shift may be more relative, due to an increase in high-quality research in other subspecialties decreasing the relative proportion of head and neck surgery focused work.

Furthermore, there is a significant increase in pediatric OHNS articles published in the journal. Our findings are consistent with a recent Canadian review which showed a greater absolute number of pediatric OHNS articles published over 20 years [20]. Another Canadian study demonstrated that Pediatric OHNS was heavily represented among program directors, and may be partially explained by differences in remuneration. As academic pediatric otolaryngologists are more commonly remunerated through an alternate funding plan [21], this may increase time allocated to research. As such, alternate funding plan may provide benefits to otolaryngologists who are interested in a combination of clinical, research and administrative duties.

### Trends by article type

When analyzing the article type, our findings indicate a significant increase in systematic and scoping reviews and a significant decrease in original research. Systematic and scoping reviews are of high clinical importance as they amalgamate existing studies to provide recommendations to clinicians [22, 23]. A systematic analysis of research availability within OHNS in 2010 identified that systematic reviews make up only 2% of publications [24]. In communication with the journal’s editorial office, the impact factor of the journal has been relatively consistently increasing over the last 9 years, from 1.020 in 2014 to the journal’s most recent 2-year impact factor of 4.856 [25]. This highlights the acceptance of higher quality scientific submissions with correspondingly higher citation and reach.

Even though original research decreased significantly, it still represents the highest absolute proportion of article type in the journal over the 9-year period. Wasserman et al. identified 75% of clinical research in their review of articles in 2006 amongst 4 major OHNS journals [26]. In comparison, our findings indicate a higher baseline and recent proportions. Clinical original studies are of vital importance for evidence-based practice. Therefore, researchers and clinicians should be encouraged to publish clinical evidence to increase the quality and quantity of original research.

### Trends in Canadian-international collaborations

Our findings indicate a significant decrease in Canadian-International collaborations with Canadian authors spearheading the publications as first or senior authors. The benefits of international research collaborations include access to additional resources, expertise, new perspectives and increased networking with others in the field [27]. On the other hand, some of the challenges of international collaborations include cultural factors (e.g., language and cultural norms), structural factors (e.g., leadership and recognition and reward systems) and professional factors (e.g., methodological preferences, work styles and past experiences) [28].

Despite the challenges, there is an increase in demand for international collaborations within the Canadian OHNS community. University of Toronto OHNS faculty’s recent strategic plan identified partnership and collaboration as one of the top five pillars with goals of promoting national and international partnerships [29]. Our findings indicate an opportunity for improved international collaborations. Clinicians spearheading research projects should be encouraged to include international collaborators to enrich research outcomes. Furthermore, the constraints of COVID-19 on international collaborations might influence our findings as well. Further research is required to track the evolution of international partnerships within the Canadian OHNS landscape.

### Trends in international publications

Our findings indicate a significant increase in both first and senior international author representation within the journal. Notably, there is a higher representation from China and United States. These findings are very encouraging as they suggest a shift from a Canadian-focused journal to one that is gaining attention in the international OHNS stage. The journal offers article processing charge (APC) waivers to papers whose corresponding authors are based in countries classified by the World Bank as low-income economies [25]. Additionally, researchers affiliated with BioMed Central’s member institutions are eligible to receive discounts on APCs in addition to funding and support services. By minimizing the financial barriers to publications, the journal provides a promising venue for researchers from developing nations. Furthermore, the journal is able to provide users with a global understanding of the current research within OHNS. Attracting high-quality international research can also help bolster the journal’s impact factor and growth. International authors may be more inclined to publish in a reputable North American journal due to financial incentives, personal credibility, career promotion and institutional credibility [30]. Furthermore, increased international publications promotes diversity within the journal and advances research visibility for users.

### Limitations

Our study is subject to a number of limitations that must be considered in the interpretation of the data. Due to the nature of information obtained, we were unable to confirm the accuracy or validity of the data collected. Finally, we also acknowledge that Canadian authors have the option of publishing with many journals nationally and internationally. Our data is limited to one journal which may affect the generalizability of the overall Canadian OHNS publication outcomes.

## Conclusion

OHNS research has undergone impressive development in the last 2 decades. The present study highlights the trends of publications within the Canadian *Journal of Otolaryngology-Head and Neck Surgery.* Our findings with respect to author characteristics decrease in MD first author contributions with a corresponding increase in medical students as first authors, and an increase in MD senior author contributions. In terms of content, we identified a significant decrease in education and head & neck related articles, increase in systematic and scoping reviews along with a decrease in original research articles published. Finally, Canadian-International collaborations demonstrate a significant decrease notably in the past 3 years of analysis. However, publications accepted from international countries are significantly increased indicating a growing global research content within the journal. Our findings shed light into the evolution of the journal since its online establishment. Further research is needed to continue characterizing the changes within OHNS literature both nationally and internationally.

## Data Availability

The datasets used and/or analyzed during the current study are available from the corresponding author on reasonable request.

## Abbreviations

OHNS: Otolaryngology—Head and Neck Surgery
EBM: Evidence based medicine
MD: Doctor of Medicine
BSc: Bachelor’s degree
MSc: Master’s degree
DDS: Doctor of Dental Surgery
Ph.D.: Doctor of Philosophy
APC: Article processing charge
